# Identification of Uterine Microbiota in Infertile Women Receiving *in vitro* Fertilization With and Without Chronic Endometritis

**DOI:** 10.3389/fcell.2021.693267

**Published:** 2021-08-13

**Authors:** Weijun Chen, Kehong Wei, Xia He, Jing Wei, Lijuan Yang, Lin Li, Tingtao Chen, Buzhen Tan

**Affiliations:** ^1^Department of Obstetrics and Gynecology, The Second Affiliated Hospital of Nanchang University, Nanchang, China; ^2^The Reproductive Hospital of Jiangxi University of Traditional Chinese Medicine, Nanchang, China; ^3^Institute of Translational Medicine, Nanchang University, Nanchang, China; ^4^School of Life Sciences, Nanchang University, Nanchang, China

**Keywords:** uterine microbiota, *in vitro* fertilization, infertility, chronic endometritis, high-throughput sequencing

## Abstract

*In vitro* fertilization (IVF) is an important assisted reproductive technology in treating infertility, whose failure rate is still high. Studies suggested that uterine microbiota are related to women’s reproductive diseases and persisting intrauterine bacterial infectious conditions, such as chronic endometritis (CE), impairing the pregnant processes. However, the relationship between uterine microbiota and IVF outcomes is still an open question. In the present study, 94 patients diagnosed with infertility were enrolled and were divided into CE (E group, *n* = 25) and non-CE (NE group, *n* = 69) groups depending on the hysteroscopy and immunohistochemistry. Subsequently, E (Ep, *n* = 8 and Enp, *n* = 17) and NE (NEp, *n* = 41 and NEnp, *n* = 28) groups were divided into pregnancy and non-pregnancy groups depending on the IVF outcomes, respectively. The uterine fluids were collected and microbial profiles were examined through the V4 region of 16S rRNA gene high-throughput sequencing. The results demonstrated that patients with CE had significantly lower clinical pregnancy rate compared with the non-CE patients (32 vs. 58.42%, *p* = 0.0014). The relative abundances of Proteobacteria and Acidobacteria were higher in the non-CE group, whereas high abundances of Actinobacteria and Fusobacteria were observed in the CE group at the phylum level. At the genus level, high relative abundances of *Gardnerella* were observed in the CE group and non-pregnancy groups, which significantly referred to the negative IVF outcome. In conclusion, CE may be a key factor for the negative outcome after IVF, of which the uterine microbiota plays a pivotal role, and the microbial diversity in uterine may serve as a biomarker to forecast the success of IVF outcome.

## Introduction

Infertility is defined by failure to establish a clinical pregnancy after 12 months of regular and unprotected sexual intercourse, and *in vitro* fertilization-embryo transfer treatment (IVF-ET) is increasingly being used in treating it (Vander Borght and Wyns, 2018). As the most important way of assisted reproductive technology, the pregnancy rate of infertile patients after IVF is about 60%, which is still not ideal, and some couples undergo repeated IVF failure (10–20%), thus heavily increasing the economic and psychological burdens of patients (Margalioth et al., 2006; Coughlan et al., 2014). The risk factors of IVF failure include advanced maternal age, smoking status of both parents, stress levels, and endometrial situation. Uterine is crucial for developing a healthy placenta, and subsequently a healthy pregnancy, thus playing a much more relevant role than the other recognized factors and deserves more exploration (Moreno et al., 2016; Bashiri et al., 2018; Puente et al., 2020).

With emerging researches on the microorganisms that existed in the human body in recent years, the traditional concept of the “sterile womb” is highly debated. The paradigm shift toward the notion that the presence of a certain microbial profile does not harm reproduction as well as contributes to a crucial process in the implantation and pregnancy is started (Benner et al., 2018). The microbial communities of the female reproductive tract have gradually become well known in pathological shifts affecting the reproductive processes, including fertilization, implantation, maintenance of pregnancy, and microbial colonization of the newborn, and are responsible for other gynecological conditions thanks to next-generation sequencing (Franasiak and Scott, 2015; Skafte-Holm et al., 2021). This adds a new microbiological dimension to human reproduction, which opens a new microbiological field in female reproductive conditions (Franasiak and Scott, 2015; Campisciano et al., 2020; Riganelli et al., 2020). Uterine is an immunologically suited niche for microbiota (Benner et al., 2018), which constitutes a physiological factor in the complex uterine environment and is dominated by Firmicutes, Bacteroidetes, Proteobacteria, and Actinobacteria, whose balance is beneficial for the maintenance of microenvironment modulating host immunity and contribution of uterine health (Cowling et al., 1992). Contrarily, altered microbial composition can have an implication for gynecological diseases, reproductive failure, and is prone to tumorigenesis (Agostinis et al., 2019; Carosso et al., 2020). Moreover, fertile endometrium benefits from the microbial contribution to physiological processes, such as endometrial receptivity, and previous literatures had pointed out the association between uterine microbial composition in terms of stability and composition and the success of reproductive process (Moreno et al., 2016). Nevertheless, the study of uterine microbiota was still limited by the vaginal contamination and low biomass, and limited study focused on the association of uterine microbiota and the IVF outcomes highlighting the healthy and unhealthy microbial compositions.

The important disease that threatens the peaceful relationship by disrupting the balance of microbial niche and immune system is chronic endometritis (CE), which is an infectious disease defined as the persistent inflammation of the endometrial lining (Moreno and Franasiak, 2017). In addition, 14–41% CE patients and 8–28% CE patients were found in patients with repeated implantation failure and repeated pregnancy loss, respectively, indicating the association of CE and pregnancy failure, but it is often asymptomatic and ignored in the clinic (Quaas and Dokras, 2008; Park et al., 2016; Moreno et al., 2018). Dysbiotic state of CE can impair entailed inflammation and immune activation in the endometrium and impact the receptivity. Specifically, immune cells (natural killer cells, dendritic cells, antigen-presenting cells) and cytokines dysregulate decidualization and thus impair the implantation process through altering vascular characteristics (vascular density vessels, endometrial proliferation, swelling), autophagy, uterine contraction, enhancing B cell extravagant and impairing endometrial receptivity via upregulating the recombinant human insulin-like growth factor binding protein-1 (IGFBP-1), and affecting maternofetal tolerance (Kitaya and Yasuo, 2010; Di Pietro et al., 2013; Franasiak, 2019). This indicates that the interactions between the host, microorganisms, microbial components, and the environment represent multiple potential consequences responsible for CE and impair reproduction (O’Callaghan et al., 2020; Xu et al., 2020).

Considering the high incidence of infertility, high failure rate of IVF, CE, which has dysbiosis of uterus cavity microbiota, and its role in infertility, we selected infertile patients with CE to represent the microbiota-dysbiosis group and relatively healthy group without CE to outline the relationship between uterine microbiota and the consequences of IVF via evaluating the pregnancy rate and uterine microbiota, which may provide insights on assessing and treating infertility couples and has significant meaning for infertility couples in the clinic.

## Materials and Methods

### Study Design and Ethical Approval

**FIGURE 1:**
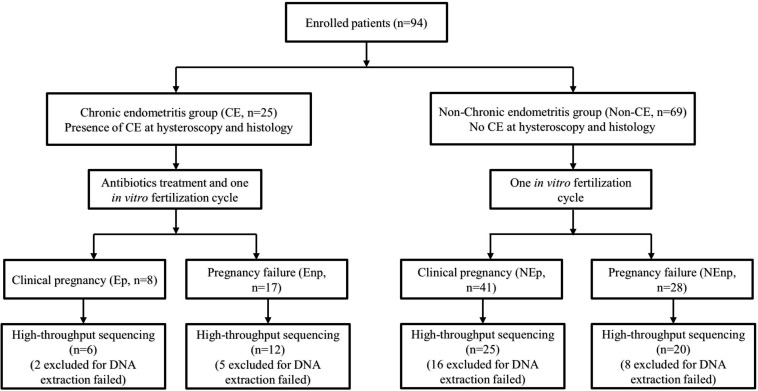
Flow diagram of the trial. IVF, *in vitro* fertilization.

This trial included studies from January 2020 to September 2020 in the Reproductive Hospital of Jiangxi University of Traditional Chinese Medicine. A total of 94 women, aged 20–38 years, were enrolled into the trial. The inclusion criteria included (1) married and had regular sex life without contraception for more than 1 year, (2) no chronic abdominal pain or lumbosacral pain, (3) had not used antibiotics for more than 1 month, (4) had never used vaginal medications and probiotics, and (5) infertility that was diagnosed without certain reason. Also, the exclusion criteria included the following:(1) had other uterine cavity diseases, including uterine cavity adhesions, submucosal uterine fibroids, uterine mediastinal tumors, and other uterine malformations; (2) had endometriosis, ovarian tumor, fallopian tube effusion, abnormal female hormones, or unexplained bleeding; (3) patients with abnormal vaginal discharge examination, such as bacterial vaginosis, *Candida albicans* infection, and *Trichomonas* infection; (4) gynecological examination that showed uterine or appendage tenderness, and suspected pelvic inflammatory disease. Diagnosis of CE was based on the histological examination of uterine tissue, and the other non-CE women were selected as another group. Patients identified with CE were undergone a 14-days course treatment depending on empirical medication. CE patients were given 250 mg ceftriaxone intramuscularly, and doxycycline (100 mg/time) and metronidazole (400 mg/time) were administered orally twice a day. All 94 women were received one IVF cycle and the CE and non-CE groups were subsequently divided into CE pregnancy group (Ep group, *n* = 8), CE non-pregnancy group (Enp group, *n* = 17), non-CE pregnancy group (NEp group, *n* = 41), and non-CE non-pregnancy group (NEnp group, *n* = 28) depending on the IVF consequences (Figure 1).

Ethical approval was obtained from the Medical Ethics Committee of Nanchang Reproductive Hospital (the Reproductive Hospital of Jiangxi University of Traditional Chinese Medicine) (approval number 2020.005). All patients signed the written informed consent, and the whole processes were in accordance with the requirements of the committee.

### Hysteroscopy

Hysteroscopy was done 3–5 days after the secretory phase. Vulva was disinfected with iodophor 1 min three times. Vagina was washed with 500 ml 0.9% sodium chloride solution. Hysteroscopy was done after the fixing of cervix. The characteristics of uterine cavity were recorded. Also, suitable endometrial tissues were scraped for subsequently pathological examination and immunohistochemistry (IHC) of CD38 and CD138/Syndecan-1 for testing the presence of plasma cells.

### Immunohistochemistry

The uterine tissue was fixed in 4% paraformaldehyde, embedded in paraffin, and sectioned. The sections were dehydrated and washed three times. IHC was performed using the anti-CD38 (Maixin Biotech, Fuzhou, China, 2010140755e) and anti-CD138 (Maixin Biotech, 1015201016) antibodies according to the standard protocols. Positive CD38 or CD138 were considered if they presented clear and complete brown staining, with complete cellular morphological characteristics. The CE was defined by the presence of one or more plasma cells under one high-power field (HPF).

### Uterine Microbiota Sample Collection

Vulva was disinfected with iodophor 1 min three times. Vagina was washed with 500 ml 0.9% sodium chloride solution. The 1.75 × 100 mm intrauterine insemination catheter devised with 1 ml injector was placed into the patient’s uterine cavity, and the uterine cavity fluid was extracted. During the process of intubation, the head of insemination catheter was inhibited from touching the vulva and vaginal wall. The uterine cavity fluid was transferred into a 1.5-ml EP tube, added with 0.5 ml saline, and centrifuged (8,000 rpm, 5 min). After centrifugation, the supernatant was aspirated and discarded, and the remaining specimens were stored in a refrigerator at −20°C until DNA extraction. Previous studies demonstrated that during a menstrual cycle, endometrial microbiota is changing with the changing of hormones, so all the samples in different groups were sampled 3–5 days after the secretory phase (CE group was sampled before antibiotic treatment) (Peric et al., 2019).

### *In vitro* Fertilization Protocol and Reproductive Outcome Analysis

Patients were given leuprolide acetate microspheres for injection (Bezon Pharm, Shanghai, 200512) downregulation therapy during 2–3 days (D2–D3) of the next menstrual cycle or directly given ovulation induction therapy. After ovulation induction therapy, 112.5–225 U recombinant human follitropin injection (Merck Serono S.P.A, BA059244) therapy was subcutaneously given during 2–3 days (D2–D3) of menstrual cycle for 10–12 days. Patients whose vaginal diameter ≥ 16 mm and dominant follicle ≥ 60% were injected with 250 μg recombinant human choriogonadotropins-ping alfa solution (Merck Serono S.P.A, BA061210) subcutaneously and underwent ovum retrieval 36–48 h later. Eggs and sperms were cultured after ovum retrieval, and the cultured embryos were transplanted. After transplantation, dydrogesterone tablet (Abbott Healthcare Products B.V., 362308) was given (10 mg/time, 3 times/day) orally, and progesterone sustained-release vaginal gel (Merck Serono S.P.A., C19131/C) was given vaginally for 10–12 days (90 mg/time).

Pregnancy outcomes were recorded by the β-human chorionic gonadotropin (β-hCG) in the peripheral blood and color Doppler ultrasound examination. Ten to twelve days after transplantation, concentration of β-hCG in the peripheral blood was tested, β-hCG > 15 mIU/ml without the development of a gestational sac was known as a chemical pregnancy. The β-hCG was checked again 5 days after the first check, the concentration ≥ 66% of original value indicated continuous pregnancy, and the dropped β-hCG indicated pregnancy failure. A color Doppler ultrasound examination was performed 30 days after transplantation. Clinical pregnancy was defined by the yolk sac, fetal heart, and fetal bud seen in the gestational sac.

### DNA Extraction and High-Throughput Sequencing

Samples in each group were conserved after sample collection for DNA extraction by a bead beating method using genomic DNA kits (Tiangen Biotech, Beijing, China) according to the manufacturer’s instructions. Then, the concentration and quality of extracted DNA were detected via a spectrophotometer at 230 nm (A 230) and 260 nm (A 260) (NanoDrop; Thermo Fisher Scientific, Waltham, MA, United States). Then the V4 region of the 16S ribosomal RNA (rRNA) genes of each sample was amplified using the primers (F: AYTGGGYDTAAAGNG, R: TACNVGGGTATCTAATCC), and PCR products were sequenced with the Illumina MiSeq platform (GenBank accession number PRJNA705499).

### Data Analysis

Illumina MiSeq platform was used for paired-end sequencing of community DNA fragments and the sequencing raw data was saved in fastq format. Sequences were aligned and taxonomically classified using the SILVA reference database. The analysis of amplicon sequencing variants (ASVs), defined by 100% sequence similarity, was analyzed by the DADA2. The alpha-diversities, containing Chao1 and Shannon indexes, represented richness and diversity. The Shannon indexes were calculated by Quantitative Insights into Microbial Ecology (QIIME2, 2019.4) and R software. Beta-diversity is also called the between-habitat diversity. Principal coordinates analysis (PCoA) made on Bray–Curtis dissimilarity matrix is the Classical Multidimensional Scaling, which was analyzed by QIIME2 (2019.4) and R software. R software was used to analyze amplicon sequencing variant Venn diagram. After removing the singleton and 10-ton ASVs from the dataset, the composition distributions of each sample at six classification levels of domain, phylum, class, order, family, genus, and species were calculated and visualized, and the analysis results are presented in the histograms. The taxonomic composition at the phylum and genus level was analyzed by QIIME2 (2019.4).

Statistical analysis was carried out using GraphPad Prism software version 8.0. Statistical differences were analyzed using *t*-test, one-way ANOVA (and non-parametric), and χ^2^-test. The *p*-value < 0.05 was regarded as statistically significant. Taxa with a false discovery rate (FDR)–adjusted *p*-value (*q*-value) < 0.05 were considered significant.

## Results

### Patient Enrollment and Baseline of Patients’ Characteristics

Ninety-four patients were enrolled into our study. In total, 25 of 94 infertility patients were diagnosed with CE (26.6%, *n* = 94). The ages and body mass indexes (BMI) of the CE group and non-CE group did not have a significant difference. In addition, the previous pregnancy, previous birth, scarred uterus, and the vaginal cleanliness degrees of patients were matched in the CE group and non-CE group. The clinical pregnancy rate in the CE group was significantly lower than the non-CE group (*p* = 0.0014). In the CE group, 4 patients (16%, *n* = 25) were observed having chemical pregnancies, and only 8 patients underwent successful clinical pregnancy, which accounted for 32% of 25 patients. For the non-CE group, chemical pregnancies were observed in 9 patients (13.04%, *n* = 69) and clinical pregnancies were observed in 41 patients (58.42%, *n* = 69) (Table 1).

**TABLE 1:** Baseline characteristics of CE and non-CE patients.

	**CE (*n* = 25)**	**Non-CE (*n* = 69)**	***P-*value**
Age (years)	30.16 ± 1.006	30.51 ± 0.5290	0.9405
BMI (kg/m^2^)	21.28 ± 0.73	20.28 ± 0.69	0.8753
Chemical pregnancy	4	9	0.7137
Clinical pregnancy	8	41	0.0014
Previous pregnancy	19	44	0.265
Previous birth	14	25	0.0857
Scarred uterus	3	8	0.9569
Vaginal cleanliness			0.5451
I	0	0	
II	25	68	
III	0	1	

*BMI, body mass index. Values are expressed as mean ± SD. P < 0.05 was regarded as statistically significant.*

### CE Accounts for a Large Proportion of Infertility Patients

**FIGURE 2:**
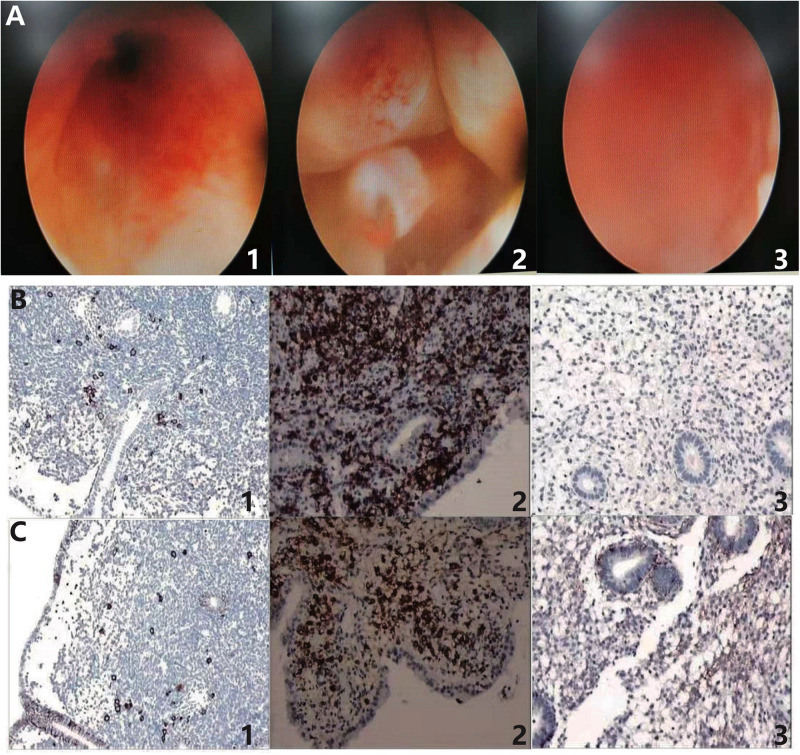
Hysteroscopy and immunohistochemical identification of plasma cells in the diagnosis of chronic endometritis. **(A_1_)** Hyperemia and **(A_2_)** micro-polyps represent the presence of inflammation in endometrium of CE patients. **(A_3_)** Negative control of non-CE patients without inflammatory endometrium. **(B_1_)** CD38 (+, > 5–18/HPF) and **(B_2_)** CD38 (+, > 20/HPF) indicate the presence of plasma cells in the endometrium of CE patients. **(B_3_)** CD38 (–) suggests the absence of plasma cell in non-CE patients. **(C_1_)** CD138 (+, > 20/HPF) and **(C_2_)** CD138 (+, > 20/HPF) also indicate the presence of plasma cells in CE patients. **(C_3_)** CD138 (–) was the negative control in non-CE patients. CE means chronic endometritis. HPF means high-power field.

The diagnosis of CE was based on the hysteroscopy and immunohistochemical identification of plasma cells. The hysteroscopy was used to identify the signs of endometrial inflammation (Kimura et al., 2019). As shown in the Figure 2A_1_, a lot of flaky, diffuse hyperemia changes could be seen, and some were punctate strawberry-like hyperemia changes, indicating the inflammation of endometrium in the CE group. Figure 2A_2_ represents the micro-polyps shown in CE patients, which showed that the neoplasms on the left and right anterior and left posterior walls of the uterus are protruding from the surface of the endometrium, and a little strawberry-like hyperemia could be seen on the neoplasms, which was the other characteristic of the inflammatory endometrium. The healthy control diagram of uterine cavity is shown in Figure 2A_3_, representing the hysteroscopy of non-CE patients, in which the endometrium was pink with no spots of flaky hyperemia, no adhesions, no vegetation, and no edema, which suggested the relative healthy uterine situation compared with the CE group.

IHC plays an important role in CE diagnosis. The representative IHC samples in the testing of CD38 are shown in Figure 2B. Figure 2B_1_ shows the positive CD38 distributed discretely in the CE patients, with > 5–18 plasma cells/HPF. There were > 20 clumped plasma cells/HPF that could be observed in Figure 2B_2_. Also, Figure 2B_3_ shows the negative control without brown particles in uterine sample of non-CE patients. CD138 is another surface antigen of the plasma cell. As shown in Figure 2C, positive CD138 samples are shown in Figures 2C_1_,_2_ in the CE patients and the negative CD138 sample is shown in Figure 2C_3_ as the negative control. In Figures 2C_1_,_2_, positive CD138 could be observed discretely and in a clumped appearance, both with > 20 plasma cells/HPF. Also, Figure 2C_3_ was the negative control in the non-CE group.

### The Alpha- and Beta-Diversities of the Uterus Cavity Microbial Community

**FIGURE 3:**
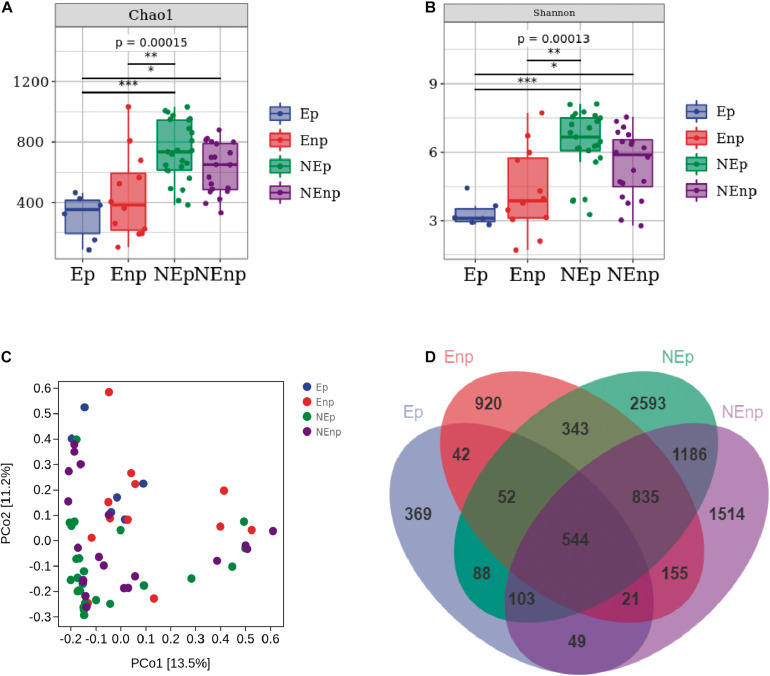
Evaluation of the uterine microbiota on alpha-diversity (within samples), beta-diversity (between samples), and Venn diagram. **(A)** Chao1 index. **(B)** Shannon index. **(C)** Principal coordinates analysis (PCoA). **(D)** ASV/OTU Venn diagram. Values are expressed as mean ± SD. *p < 0.05, **p < 0.001, ***p < 0.0001.

We analyzed a total of 63 uterine fluid samples. 16S rRNA amplicon sequencing analysis was used to sequence the V4 hypervariable region. The effective tags of all samples were clustered. In total, there were 5,940 ASVs obtained in all 63 samples and on average, there were 1,485 ASVs in each group. In total, 7,485,326 filtered clean tags were observed in 63 samples and the average in each group was 118,814.70 (data not shown), and 6,967,820 high-quality sequences were detected. As shown in Figures 3A–C, α- and β-diversities (Chao1, Shannon, and PCoA) were analyzed to estimate the diversities within and between different groups, respectively. The results showed that the Chao1 index in the CE group was lower than that in the non-CE group, indicating the richness in the CE group that was lower than the non-CE group. The diversity of non-CE group, with significantly higher Shannon index, was higher than the CE group. In addition, distribution of the microbial community showed by PCoA indicated that the samples in the Ep and Enp groups were clustered together and the samples in the NEp and NEnp groups were close to each other. However, the samples in the CE group were far away from the non-CE group. The common ASV Venn diagram suggested that there were 544 common ASVs found in all groups, and the unique ASV number in the Ep, Enp, NEp, and NEnp groups were 369, 920, 2,593, and 1,514, respectively (Figure 3D). Also, *Atopobium vaginae* was identified from the Enp group.

### Uterine Microbiota Differs in CE and Non-CE Patients and With Different Reproductive Outcomes

**FIGURE 4:**
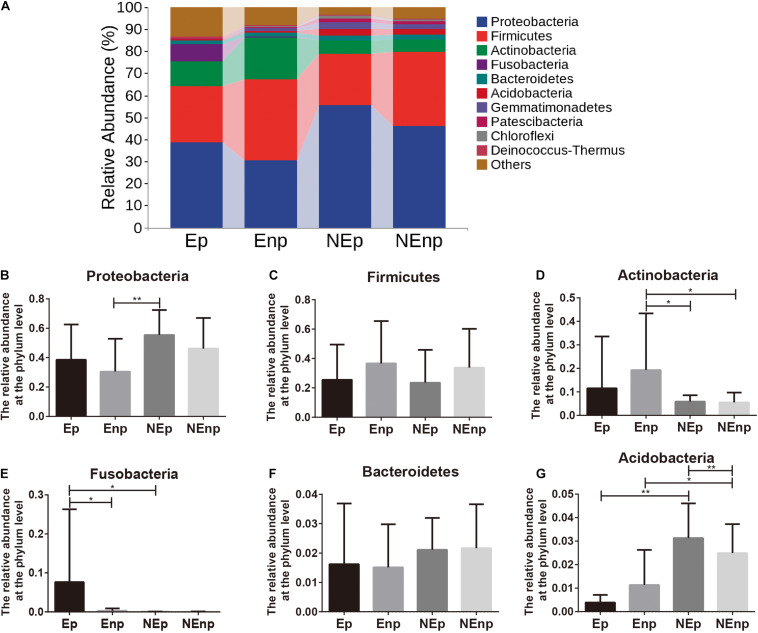
Relative abundance of uterine microbiota at the phylum level. **(A)** The relative abundances of the top 10 bacteria at the phylum level. The relative abundances of **(B)** Proteobacteria, **(C)** Firmicutes, **(D)** Actinobacteria, **(E)** Fusobacteria, **(F)** Bacteroidetes, and **(G)** Acidobacteria. Values are expressed as mean ± SD. *q < 0.05, **q < 0.001.

**FIGURE 5:**
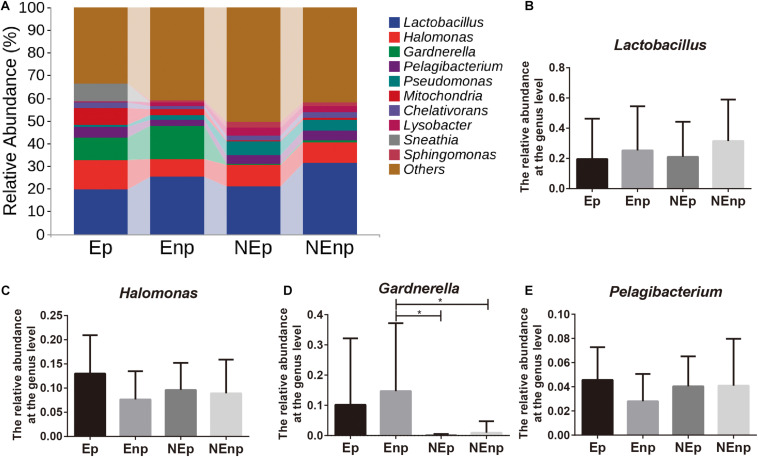
Relative abundance of uterine microbiota at the genus level. **(A)** The relative abundances of the top 10 genera bacteria at the phylum level. The relative abundances of **(B)** *Lactobacillus*, **(C)** *Halomonas*, **(D)** *Gardnerella*, and **(E)** *Pelagibacterium*. Values are expressed as mean ± SD. *q < 0.05.

The relative abundances of the top 10 bacteria at the phylum level were analyzed (Figure 4A). Proteobacteria was the most dominated bacteria in all groups, and the relative abundances of Proteobacteria in the CE group were significantly lower than in the non-CE group, which accounts for 38.54, 30.50, 55.44, and 46.16%, respectively (Figure 4B). The other prominent bacteria in the Ep, Enp, NEp, and NEnp groups were Firmicutes, Actinobacteria, Fusobacteria, Bacteroidetes, and Acidobacteria (Figures 4C–G). The relative abundances of Acidobacteria were significantly higher in the non-CE patients, and the relative abundance of Proteobacteria in patients with pregnancy outcome after IVF was higher than the non-pregnancy patients in CE and non-CE groups, respectively. In addition, Acidobacteria was higher in the pregnancy group compared with the non-pregnancy group, showing its benefit for women. The relative abundance of Actinobacteria was significantly higher in the CE patients and was higher in non-pregnancy groups. A large number of Fusobacteria was significantly identified from the Ep group. The most prominent bacteria at the genus level are shown in Figure 5A. As shown in Figure 5B, both in the groups with and without CE, *Lactobacillus* was identified as the most abundant genera, whose relative abundances were 19.56% in the Ep group, 25.28% in the Enp group, 21.08% in the NEp group, and 31.53% in the NEnp group. Among this, *Lactobacillus inners* was the most predominant species (data not shown). Also, the other prominent genera were analyzed, including *Halomonas*, *Gardnerella*, and *Pelagibacterium*, which are shown in Figures 5C–E, respectively. The relative abundance of *Gardnerella* was significantly more prominent in the CE group compared with the non-CE group, and was higher in the non-pregnancy group compared with the pregnancy group in both CE and non-CE patients.

## Discussion

We screened 94 patients and 26.6% were diagnosed with CE. The clinical pregnancy rate of the CE group was significantly lower than that of the relative healthy non-CE group (32 vs. 59.42%, *p* = 0.0014). The normal composition and fluctuation of immune cells in the endometrium are important for normal endometrial receptivity (Li et al., 2020). In CE patients, the B lymphocytes is significantly higher in the endometrial layer and the basal layer compared with healthy women, whose B lymphocyte infiltration rate is less than 1% of the entire leukocyte population (Kitaya et al., 2018). Changed immunocompetent cells in CE patients impair the recruitment of natural killer cells, which are responsible for the local immune reaction in early pregnancy and implantation, and explain the decreased implantation and pregnancy rates after IVF in CE patients. What is more, CE hampers the endometrial receptivity by impacting endometrial proliferation, apoptosis, sex hormone production, trophoblast cell invasion, and decidualization, which is a progesterone-dependent process and is important for implantation, and ultimately leads to reproductive failure, especially in patients who underwent IVF (Puente et al., 2020). The significantly different clinical pregnancy rates between CE and non-CE patients indicated that the microbiota in the uterus cavity propose more complex effects on IVF.

Female reproductive system harbors 9% of total human colonized microbiota. Today, the challenge for scientists is to understand the hidden causes of infertility and improve the efficiency and effectiveness of IVF technology. Studies revealed that the upper part of reproductive system is also colonized by various microbiota. However, the peritoneal fluid and endometrium contains 10,000 times less bacteria when compared with the vaginal microbiota (Chen et al., 2017). For uterus, researches agreed that Firmicutes, Bacteroidetes, Proteobacteria, and Actinobacteria account for the main phyla, which is coincident with our results (Cowling et al., 1992). Our results demonstrated that the relative abundance of Proteobacteria was higher in the non-CE group and successful pregnancy groups, which indicated that Proteobacteria is a beneficial bacterium. The relative abundances of Firmicutes and Bacteroidetes did not show much difference in different groups, and one previous paper indicated that Bacteroidetes is the core bacteria of the uterine microbiota in non-pregnant women (Verstraelen et al., 2016). However, Actinobacteria, the opportunistic pathogen, was higher in the CE group and non-pregnancy groups significantly, and was related to adverse IVF outcome. Fusobacteria had a negative impact on the pregnancy rate (Pelzer et al., 2013). Although it was not specifically discussed in our study, *Fusobacterium nucleatum* is associated with the periodontitis and pregnancy diseases (Cobb et al., 2017). CE patients had uterine dysbiosis, and one theory suggested that the changed microbiota can increase the host sensitivity to immune system, trigger inflammation process, disturb immune responses, and impair endometrial receptivity, and thus subsequent reproductive processes (Bardos et al., 2019).

In the research on the female reproductive tract microbiota, *Lactobacillus* accounts for a large attention, especially in the vagina (Chen et al., 2017). Our study suggested that *Lactobacillus* was the most prevalent genera in uterine, but did not directly relate to the IVF outcomes. This concept is supported by studies focused on the endometrial microbiota at the term of pregnancy claiming that ongoing pregnancies in women with an endometrium have a complete lack of *Lactobacillus* (Hashimoto and Kyono, 2019). Research collected endometrial microbiota samples directly after transabdominal hysterectomy and suggested that *Lactobacillus* was detected in less than 1% in endometrial samples, and Winters et al. (2019) also indicated that endometrium is not dominated by *Lactobacillus*, whose relative abundance is variable when the uterine fluids were not collected transcervically (Franasiak and Scott, 2015; Leoni et al., 2019). High relative abundance of *Gardnerella* was presented in the group with low clinical pregnancy rate, which indicated the adverse effects on pregnancy outcome, and *vice versa*. In addition, the relative abundance of *Gardnerella* in the pregnancy group was lower compared with the non-pregnancy group both in CE and non-CE patients, respectively. These results suggested that *Gardnerella* was responsible for CE and low pregnancy rate and previous studies also suggested that its high abundance is associated with endometritis, endometriosis, and the low rate of pregnancy (Swidsinski et al., 2011). These results and our significantly low abundance of *Gardnerella* in the non-CE groups together suggested that *Gardnerella* is the unwanted bacteria in the uterus and is related to the low pregnancy rate in both natural and IVF processes (Onderdonk et al., 2016). *Atopobium vaginae*, which is a Gram-negative bacteria and can produce endotoxin and stimulate pro-inflammatory cytokine production, was particularly identified from the Enp group, indicating the especially negative impact on reproductive result after IVF. In addition, the main component of the outer membrane of Gram-negative bacteria, lipopolysaccharide, increases the chemokine (C-X-C motif) ligand 1 protein (CXCL1), CXCL13, and selectin E level in the uterine, and sustains the inflammation and recruits B cells, which is the mediator of CE and indicates endometrial dysfunction, and then leads to architectural changes and focal stromal breakdown (von Hundelshausen et al., 2017). That is, the uterine microbiota exert effects on endometrium through modulating gene expression, altering epithelial cell permeability, and changing the balance between pro-inflammatory and anti-inflammatory reactions (Molina et al., 2020).

Importantly, commensal uterine microbiota enable the endometrium suited for healthy pregnancy and unhealthy endometrial microbial composition can explain the infertility with uncertain reasons and the low pregnancy rate after IVF. Also, it is possible that doctors can predict the pregnancy outcome of couples by identification of the microbiota of reproductive system, which can decrease the economic and psychological burdens of patients of IVF failure. The intervention of microbiota of the female reproductive system had shown benefits on reproductive diseases, such as bacterial vaginosis. Also, these results give us an insight that we can promote the consequence of fertility and IVF by providing correlated microbial intervention, including probiotics, prebiotics, and microbial transplantation, to alter the composition of endometrial microbiota before the next attempt for conception, which give us a new way for the treatment of infertility and improve the IVF outcome. Some limitations still exist in the current study, including the number of subjects involved, sequencing technique, and sampling method. The ethical approval and enrollment were limited by the specific patients who are infertile and needed IVF. More proper sampling methods should be given to avoid the contamination of vaginal microbiota. In further studies, animal experiments and integrated -omics studies will be used to reveal the more underlying mechanisms.

## Conclusion

Twenty-five in 94 infertile asymptomatic patients were diagnosed with CE and the clinical pregnancy rate after one IVF cycle of CE patients was significantly lower than that of the non-CE patients. The relative abundances of Proteobacteria and Acidobacteria were higher in the non-CE group. In addition, Acidobacteria was higher in the pregnancy group. Actinobacteria and Fusobacteria were higher in the CE group at the phylum level and Fusobacteria were the risk factor for IVF failure. In addition, the relative abundance of the *Gardnerella* in the CE group was higher than in the non-CE group, and was higher in the non-pregnancy groups. These results confirmed the microbiota-colonized uterine environment and suggested that the dysbiosis is negative to the IVF consequences. This may provide new possible causes for IVF failure and may provide a new clinical management direction for infertile couples and an adjunctive treatment for women undergoing IVF by restoring a favorable uterine microbiota. More underlying mechanisms are also needed to be clarified in the future.

## Data Availability Statement

The datasets presented in this study can be found in online repositories. The names of the repository/repositories and accession number(s) can be found in the article/supplementary material.

## Ethics Statement

The studies involving human participants were reviewed and approved by the Medical Ethics Committee of Nanchang Reproductive Hospital. The patients/participants provided their written informed consent to participate in this study.

## Author Contributions

TC and BT conceived and designed the study. WC performed the experiments. WC, KW, XH, and JW analyzed the data and wrote the article. LY and LL generated the figures and tables. All authors contributed to the article and approved the submitted version.

## Conflict of Interest

The authors declare that the research was conducted in the absence of any commercial or financial relationships that could be construed as a potential conflict of interest.

## Publisher’s Note

All claims expressed in this article are solely those of the authors and do not necessarily represent those of their affiliated organizations, or those of the publisher, the editors and the reviewers. Any product that may be evaluated in this article, or claim that may be made by its manufacturer, is not guaranteed or endorsed by the publisher.
